# Paternal response to novel predator exposure correlates with transgenerational response in offspring of threespined stickleback

**DOI:** 10.1098/rsos.240878

**Published:** 2025-01-08

**Authors:** Michaela M. Rogers, Jennifer K. Hellmann

**Affiliations:** ^1^ Department of Biology, University of Dayton, Dayton, OH 45469, USA; ^2^ Department of Evolution, Ecology, and Organismal Biology, The Ohio State University, Columbus, OH 43210, USA

**Keywords:** *Gasterosteus aculeatus*, multimodal signalling, paternal effect, phenotypic plasticity, predation, transgenerational plasticity

## Abstract

Parental experiences can alter offspring phenotypes via transgenerational plasticity (TGP), which may prime offspring to adaptively respond to novel stressors, including novel predators. However, we know little about the types of sensory cues (e.g. visual, olfactory) that parents use to recognize novel predators and the consequences for offspring. Individuals may respond to novel cues if they mimic historical cues or they may need multiple sensory cues to recognize and respond to novel stimuli. We exposed threespined stickleback (*Gasterosteus aculeatus*) males to a full factorial of visual and olfactory cues of a novel trout predator prior to fertilization and tested offspring for antipredator behaviour and survival against a live predator. Fathers exposed to visual cues oriented more to and spent time closer to the novel predator post-exposure on the first day. Paternal response to visual cues was echoed in their offspring: offspring of fathers exposed to visual cues were caught faster by a live predator, suggesting that multiple cues are not needed to induce a transgenerational effect. While visual cues elicited responses both within- and transgenerationally, they do not seem to result in adaptive priming in offspring, suggesting the possibility of maladaptive TGP in response to novel cues of predation risk.

## 1. Introduction

Phenotypic plasticity is when an individual’s experiences during its lifetime influence its phenotype (e.g. behaviour, morphology and physiology) in ways that may allow it to respond to rapid environmental changes [1,2]. For example, free-ranging nightingales adjusted their territorial song amplitude in response to the amount of environmental background noise [3]. In addition to experiences within their lifetime, organisms can also learn about their environment through their parents, via transgenerational plasticity (TGP). TGP occurs when experiences in the parental environment influence the behaviour, morphology and/or physiology (i.e. phenotype) of future generations [4]. In crickets, gravid females that were exposed to a predator had offspring that exhibited increased antipredator behaviours and increased survival against a predator compared to offspring of control mothers [5]. These parental cues reflect parents’ experiences prior to or around the time of fertilization, including experiences early in life [4]. These cues can be passed to offspring through a variety of mechanisms and at different life stages, ranging from the time of fertilization (e.g. DNA methylation, histone modification, sperm and egg RNA, hormonal composition in eggs) to post-emergence (e.g. behavioural changes in parental care behaviour) [4]. Through these mechanisms, parents can prime their offspring for the environments they are going to encounter, which is particularly adaptive when parent environments are highly predictive of offspring future environments [6]. This was shown in a coral reef fish, where offspring were able to adapt to rising temperatures if their parents had first been acclimated to those temperatures [7].

However, when parents receive novel cues (e.g. cues from a non-native predator), the ability to predict future environments and respond appropriately becomes more difficult. Parents could fail to recognize the cue as dangerous, especially if the cue is dissimilar to any current environmental cues [6]. This could happen, for example, because novel predators emit novel cues [8], potentially making it difficult for prey to pair a novel cue with an indication of danger. If the novel cue is misinterpreted, it could cause maladaptive parental effects when the resulting offspring phenotype does not suit the changing environment [6]. However, individuals may be able to learn about novel cues in their environment when they are associated with a known cue [9]. For example, when a parent sees a novel organism eat a conspecific and/or detects the presence of conspecific damage cues, this likely indicates that this novel organism is a predator. In this case, even novel cues may elicit a stress response in parents (e.g. via activation of the hypothalamic–pituitary–adrenal axis) that can have consequences for offspring. Recognizing novel cues may be especially likely if pathways underlying plasticity, including TGP, have evolved to be generalized, such that TGP is elicited in response to any cue that indicates a low-quality environment. There is evidence of this: for example, multiple environmental stressors influenced corticosterone levels in western bluebird mothers which in turn influenced aggression levels in their sons [10]. Alternatively, there is evidence to suggest that transgenerational effects may be specific to the stressor experienced by the parental generation. For example, Chen *et al*. found that transgenerational responses to native predators were different from responses to a novel stressor [11].

The type of sensory cue encountered by parents may affect how easily and accurately parents can detect changes in their environment, and therefore the adaptive nature of these effects (i.e. how well the phenotype of the offspring fits the ideal phenotype for the environment). Certain types of novel sensory cues (e.g. visual, olfactory) may be easier to recognize as dangerous compared to others. This is because visual and olfactory cues transmit different information about the environment. Visual cues can reveal information about the presence of a predator, including the position and/or distance; however, the absence of visual cues indicates that predators are absent from the immediately surrounding area but not the entire habitat. Meanwhile, olfactory cues could potentially be a better indicator that predators are absent from the entire habitat because these cues persist even after the predator has left the immediate area [12]. Many aquatic species rely on olfactory cues over visual cues to assess predation [13] and only use visual cues when chemical information is missing or ambiguous [14,15]. Olfactory cues could provide better information about the future environment, especially if olfactory cue concentration detected in the environment can be scaled to the density of predators [16]. However, aquatic species may switch from chemical to visual cues depending on the characteristics of the predator (e.g. whether the predator is stationary or mobile [17,18]) or the body of water (e.g. static versus flowing water [19]). Even though prey may typically respond to one cue type over another, they may still have the capability to respond to both cues depending on the context [12].

When cue reliability is decreased as in the case of novel cues, having multiple sensory cues may be advantageous to increase the certainty about the potential danger in the environment and provide a redundant signal that allows receivers to respond more accurately to a situation (redundant signal hypothesis [20,21]). However, multiple types of cues have the potential to interact with each other to alter the response of the receiver [20]. Perch that were presented simultaneously with both visual and olfactory cues of a predator reduced their foraging more drastically than when they received each cue alone, suggesting that having multiple cues could have an additive or even synergistic effect [22]. We generally lack information about how individuals respond when multiple cues are received simultaneously and how offspring respond when this information is passed transgenerationally. Knowing the types of cues parents rely on to inform their offspring of predators in the future environment allows for a greater understanding of how populations may cope with rapid human-induced changes such as invasive predators.

To investigate how parents respond to novel cues, we exposed threespined stickleback (*Gasterosteus aculeatus*) fathers to visual, olfactory, or a combination of both cues from a novel rainbow trout predator (*Oncorhynchus mykiss*) for 10 days and compared their behaviour before and after the addition of the cues on days 1, 5 and 10. While trout is a novel predator for this population of sticklebacks, we know from previous studies that threespined stickleback are able to quickly respond to cues of a rainbow trout predator and increase their antipredator behaviour after exposure to trout cues [23]. Sticklebacks are also considered primarily visual fish [19], with previous evidence showing they respond more to visual cues when compared to olfactory cues [24]. Despite that, additional evidence shows they are still able to distinguish predators from non-predatory fish using olfactory cues alone [25]. Sticklebacks are also an excellent system for exploring responses to novel cues, given their repeated and independent radiation to freshwater environments in which they often encountered novel environments, including the presence of novel predators [26,27]. We predicted that if fathers recognized the cue to be associated with a predator, then they would respond by increasing their antipredator behaviour after receiving the cue, such as moving further away from the predator, decreasing time spent in the nest, and increasing hiding compared to control individuals [28].

To understand how novel cue types affect offspring development, we generated offspring from fathers exposed to visual cues of a novel predator, olfactory cues of a novel predator, or both. Predator-induced maternal and paternal effects are well documented in sticklebacks, including sperm-mediated paternal effects. For example, fathers exposed to predation risk prior to fertilization produced offspring that were less anxious and exhibited more risk-taking [29,30]. We measured offspring activity in an open field assay and survival against a live predator when they were 5 months old to ensure that they were large enough to be handled but small enough to be consumed by the predator. We predicted that if fathers showed stronger behavioural responses to one sensory cue compared to the other (e.g. attend more strongly to visual cues of predation risk [24]), then there would be a stronger TGP response in their offspring. In other words, we predicted that the magnitude of the paternal response would indicate the future magnitude of F1 responses. We also predicted that, if fathers responded more strongly to receiving both visual and olfactory cues simultaneously, then we would expect stronger transgenerational effects in offspring of fathers who received both cues compared to offspring of fathers who only received visual or olfactory cues independently. Finally, we predicted that if paternal exposure to the visual or olfactory cue was adaptive for offspring, then offspring would be less active in the open field assay [11,31], and they would not be captured as quickly by a live predator if their father was exposed to that cue [32,33].

## 2. Methods

### 2.1. Housing/sourcing

We collected adult threespined stickleback (*Gasterosteus aculeatus*) from Putah Creek (CA, USA) in June 2021 and shipped them to the University of Dayton (OH, USA). For two months prior to the start of the exposures, they were housed on a recirculating system in groups of 10–12 fish per tank to mimic shoaling conditions in the wild. The system was maintained on a summer photoperiod schedule (16L:8D) at 20 ± 1°C. We fed the fish twice a day ad libitum with a mixture of frozen bloodworms (*Chironomus* spp.), brine shrimp (*Artemia* spp.), Mysis shrimp and Cyclop-eeze. We obtained rainbow trout (*Oncorhynchus mykiss*) from Freshwater Farms of Ohio in June 2021. While rainbow trout are present in Putah Creek, they are not present in Beaver Pond (J.K.H. & A. Bell, personal observation), which is an isolated section of Putah Creek where we collected the stickleback. This provides an environmentally relevant yet novel predator.

### 2.2. Preparation of predator cue

To prepare the trout cue, we used methods adapted from [34]. We weighed a trout (50–70 g, 10–15 cm long) before placing it in a new 37.9 l (53 L × 33 W × 25 H cm) tank. We filled the tank with clean, conditioned tap water proportional to the size of the trout (500 ml g^−1^ of fish). The tank contained gravel and two sponge filters. We fed the trout one live juvenile stickleback per day for four days. After 96 h, we returned the trout to its holding tank and froze the tank water into 200 ml aliquots using Whirl-Pak sample bags.

### 2.3. Experimental housing conditions

**Figure 1.**
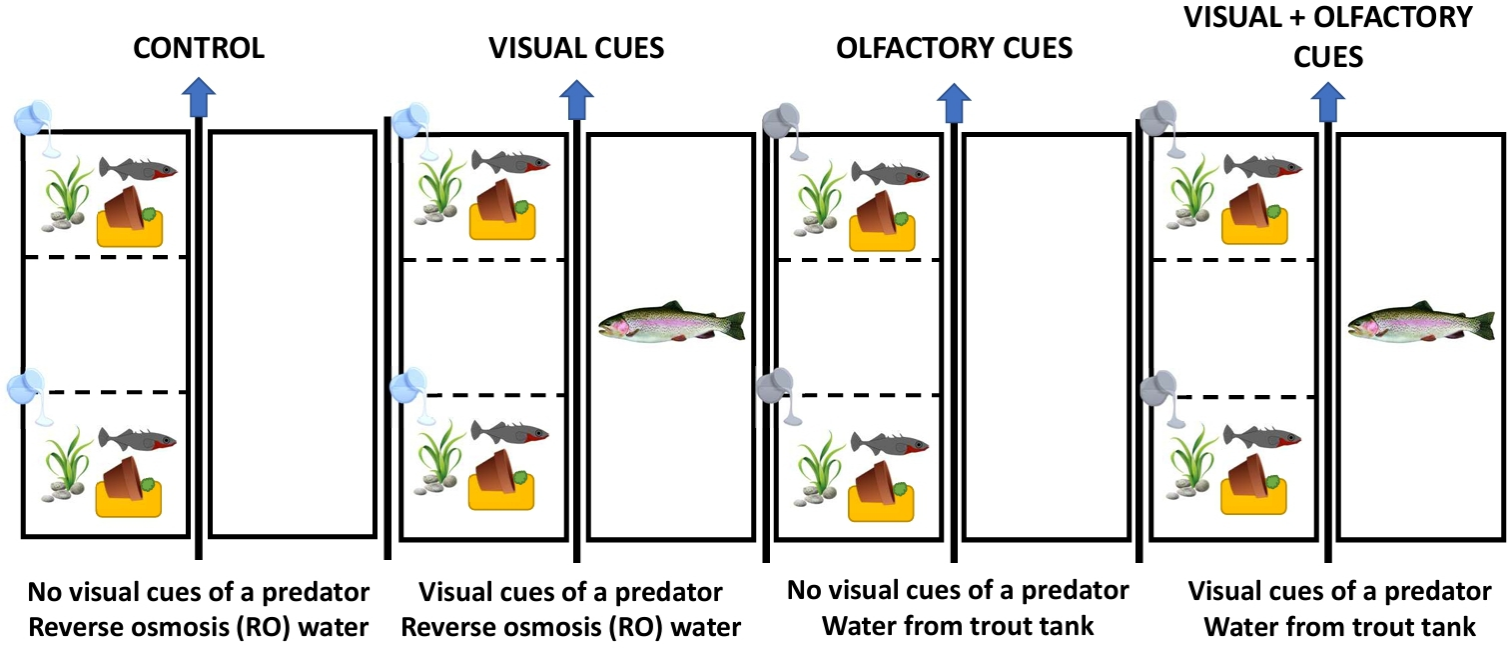
Top view of the paternal exposure set-up for each treatment. We housed two stickleback in opposite compartments of a 37.9 l tank divided into three sections and housed the trout individually in an undivided 37.9 l tank. We separated the stickleback and predator tanks visually with a removable opaque divider.

In August 2021, we moved males into experimental 37.9 l (53 L × 33 W × 25 H cm) stand-alone tanks. We divided each tank into three equal sections using removable barriers with nine 3 mm holes to allow for water flow between the sections. We placed one male stickleback in each of the outer sections and left the middle section empty. The end sections contained gravel, one fake plant and nesting materials: a container of sand, a terra cotta pot for cover and algae. The middle section contained only gravel. Next to each nesting male tank, we placed an undivided 37.9 l tank filled with freshwater and containing gravel and two sponge filters. Depending on the visual predator treatment, the tank was either left empty or contained one live rainbow trout. A schematic of the placement of the stickleback and trout tanks is shown in figure 1.

### 2.4. Exposure regime

We began exposures after at least 50% of the males had nested, which signalled that they were ready to mate. We exposed stickleback once a day for 2 h for a total of 10–14 days (figure 1) (depending on the availability of gravid females; see below). All experimental tanks were separated visually from each other with a removable opaque divider. At a random time of day between 08.00 and 20.00, we turned off the bubblers in the tanks and removed the opaque divider to reveal the adjacent tank as either empty or containing a live trout. Using a beaker, we gently poured in 100 ml of either control reverse osmosis (RO) water or the prepared predator cue to each end compartment containing one male stickleback immediately after pulling the barrier starting on day 1. In the control treatment, we removed the divider to show a tank filled with only water and we gave each fish 100 ml of RO water. In the visual treatment, we removed the divider to show a live trout in the adjacent tank and we gave each fish 100 ml of RO water. In the olfactory treatment, we removed the divider to show a tank filled with only water and we gave each fish 100 ml of the prepared predator cue. In the combination of visual and olfactory treatment, we removed the divider to show a live trout and we gave each fish 100 ml of the prepared predator cue. Every other day, we fed the trout one juvenile stickleback during the 2 h exposure to reinforce the visual cue (for both visual-only and visual and olfactory treatments). After 2 h, we replaced the dividers and completed a 50% water change on all treatments.

On days 1, 5 and 10 of exposure, we took a 10-min video before the barriers were pulled (‘baseline’) and 10 min after the first barrier was pulled (‘exposed’) to examine changes in male behaviour before and after the exposure. We did not score the first 5 min of the videos to allow for acclimation to the camera presence. We scored the last 5 min of the videos from the paternal exposures using BORIS tracking software [35]. For fathers, we scored time in the front half of the tank (nearest to the predator and open relative to the back of the tank, which contained plants and a pot), time oriented to the predator and time hiding. Time hiding was scored when the fish was behind the plant, in the plant, or under the lip of the nesting box.

### 2.5. Generating offspring

We removed males after the last day of exposure (day 11) if a gravid female was available. If a female was not available on day 11, the male would continue to be exposed once a day. *n* = 14 males were removed on day 11, *n* = 16 on day 12, *n* = 5 on day 13, and *n* = 3 on day 14. Once removed, we euthanized the male via decapitation. We dissected the testes and gently squeezed the eggs out of an unexposed female to artificially fertilize in a Petri dish. After fertilization, we transferred the fertilized eggs to egg cups with mesh bottoms over air stones until they hatched (~6 days post-fertilization [36,37]). We housed the fry by clutch and raised them without parental care from fathers in 18.9 l tanks (38 L × 25 W × 21 H) on a recirculating flow-through system. We fed offspring brine shrimp until they were old enough to eat frozen food (2–4 months old). We raised *n* = 9 control clutches, *n* = 8 visual clutches, *n* = 7 olfactory clutches and *n* = 8 visual and olfactory clutches.

### 2.6. Offspring testing

At 5 months old (January–April 2022), we individually tested offspring behaviour in an open field assay after exposure to a model trout predator. Forty-eight hours before the assay, we took standard length and mass before moving them to an individual holding tank. We also swabbed the sides of all individuals at this time to obtain a DNA sample for sexing. The behavioural arena was similar to that used in [38]. We filled a circular plastic bucket divided into eight outside wedges and a ninth circular section in the middle (12 cm diameter) with clean water (18 cm diameter, 8 cm water depth). To begin, we released the focal fish from a cup in the middle of the arena and allowed them to acclimate freely in the arena for 25 min. To measure activity and exploration, we scored the number of total sections visited and the number of different sections visited for 5 min (‘baseline’ behaviour). Then we gently poured in 200 ml of trout predator cues from a beaker (as prepared above) and chased the fish with a 10 cm model of a rainbow trout for 15 s. We measured time frozen followed by 5 min of ‘exposed’ behaviour once they resumed movement; the trial ended if the fish froze for 5 min. We did a 100% water change and reset the arena between trials. For each treatment, we assayed *n* = 59 control individuals, *n* = 57 visual individuals, *n* = 54 olfactory individuals and *n* = 64 visual and olfactory individuals (paternal cue).

After 24–48 hours of the behavioural assay, we conducted survival assays in groups of two siblings (therefore they had the same paternal treatment and genetic family). We used trout for survival assays across a two-day period. We filled four circular portable pools with conditioned tap water (1 m diameter, 13 cm water depth) and evenly placed six plants around each arena. We placed an individually marked rainbow trout in each pool 24 h before the first day of assays, which we fasted for 48 h prior to being placed in the pools. Prior to the first day of assays, we fed them one non-experimental juvenile stickleback after being placed in the pool to keep hunger levels consistent between the first and second day of assays. On the day of the assays, each trout was used for two subsequent assays, one in the morning and one in the afternoon. To begin, we placed two siblings in a clear cylinder (20.3 cm diameter) in the middle of the arena. After acclimating for 20 min, we lifted the cylinder and used a stopwatch to time how long it took the trout to capture one of the fish (survival time). We ended the assay after 1 h if the trout had not captured either prey (*n* = 2 assays); we ran *n* = 87 trials in total. Therefore, we had *n* = 85 successful assays: *n* = 26 control, *n* = 20 visual, *n* = 17 olfactory and *n* = 22 visual and olfactory.

### 2.7. Offspring sexing

We were initially interested in how offspring survival and behaviour were affected by sex so we took a DNA swab of the offspring to sex a large portion of the individuals with a genetic marker following methods from [39]. While threespined sticklebacks can be sexed genetically using a tissue sample such as a fin clip, we did not want to compromise swimming activity during assays; therefore we opted to collect mucus non-invasively. Of the 234 offspring swabbed, we were unable to determine sex for *n* = 48 individuals due to insufficient DNA samples. For all treatment groups, the sex ratio was also highly skewed with overall more females than males (electronic supplementary material, table S1). Given the missing and skewed data, we decided not to include sex as a covariate in the final models. An analysis including sex is provided in the electronic supplementary material.

### 2.8. Statistical analysis

For all paternal and offspring traits, we tested for differences in variance due to paternal treatment using Levene’s tests in R. To determine mean differences in traits due to paternal treatment, we ran MCMC generalized linear mixed models (R package MCMCglmm [40]). All models (paternal behaviour, offspring activity and offspring survival) had 200 000 iterations, a burn-in of 3000 iterations, thin = 3 and Poisson distributions with a weak prior. For paternal behaviour, we ran models for time in the front half of the tank and time hiding. Time in the front half of the tank and orientation to the predator for paternal behaviour were highly correlated so we only analysed time in the front (Spearman’s rank correlation: *ρ* = 0.79, *p* < 0.001; see electronic supplementary material, table S2, for paternal time oriented to the predator). We tested fixed effects of visual exposure (yes/no), olfactory exposure (yes/no) and observation period (pre/post) with the random effects of the observer (video scorer), male identity (paired data) and tank identity to account for pseudoreplication for males sharing a tank. Separate models were run for each observed day of exposure (days 1, 5 and 10). Given our *a priori* predictions about the interactions between sensory cues, we included interactions between visual treatment and olfactory treatment (and observation period for activity), then removed any nonsignificant interactions to arrive at the final models.

For offspring activity in the behavioural assay, we ran models for different sections visited and total sections visited with fixed effects of visual exposure, olfactory exposure, observation period and length with random effects of the observer, fish identity (to account for repeated observations), and clutch. For freezing behaviour, the model included fixed effects of visual exposure, olfactory exposure and length with the random effect of observer and clutch. For the survival assay, we ran a model for survival time using fixed effects of visual exposure, olfactory exposure and length with random effects of trout identity, assay number (to account for improvements in predator performance), and clutch. In trials where both fish were captured at the same time (*n* = 8 trials), we removed the duplicate survival time and only included one fish’s survival time for that assay. We again tested for interactions between visual and olfactory cues and removed nonsignificant interactions as necessary.

## 3. Results

### 3.1. Paternal behaviour

**Figure 2.**
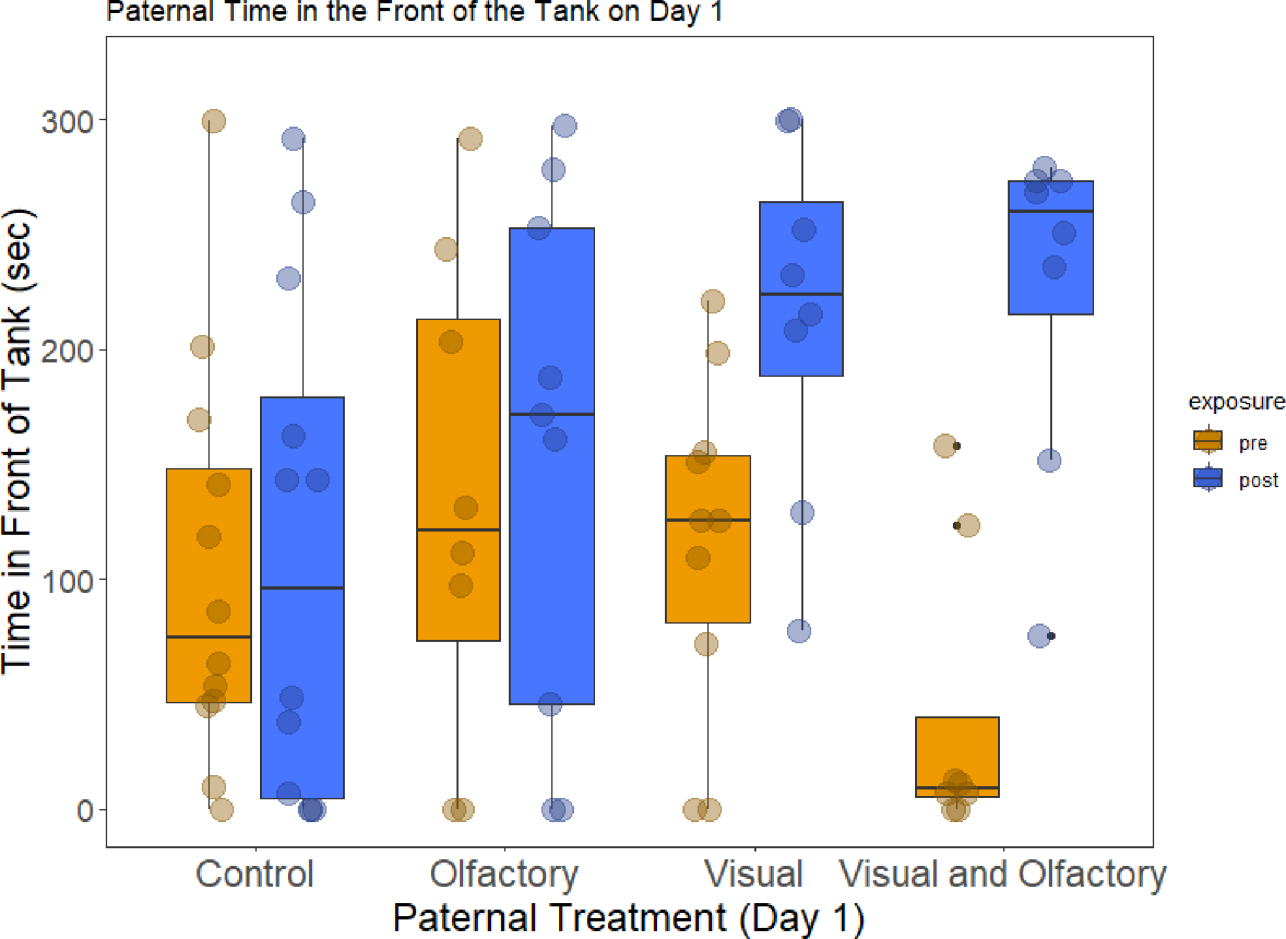
Paternal time spent in the front of the tank on day 1 due to paternal treatment (data are median with interquartile range, dots correspond to individuals). Orange boxplots show the baseline behaviour of fathers 5 min before treatments were applied. Blue boxplots show paternal behaviour 5 min after treatments were applied. We found a significant interaction between paternal visual cue exposure and the observation period on day 1 for time spent in the front of the tank. Fathers exposed to the visual cue in the visual-only or visual and olfactory treatment spent more time in the front of the tank after exposure compared to control fathers and olfactory-only exposed fathers.

We exposed males to visual and/or olfactory cues of a predator and measured their behaviour pre- and post-exposure on days 1, 5, and 10. We found a significant interaction of visual treatment and exposure period on day 1 for time spent in the front half of the tank (figure 2; table 1). Specifically, we found in the post-exposure period that fathers exposed to visual cues (in either the visual-only or visual and olfactory treatment) spent significantly more time in the front half of the tank compared to the control (MCMC GLMM, 95% CI in brackets here and below; visual treatment: [0.12, 4.83], *p* = 0.04; visual and olfactory: [0.03, 4.90], *p* = 0.05), but those differences were not found in the pre-exposure period (visual treatment: [−3.09, 2.84], *p* = 0.96; visual and olfactory: [−5.00, 1.34], *p* = 0.24). We also did not find evidence that olfactory treatment altered paternal behaviour before or after exposure on day 1 (table 1). By day 5 and day 10, we found no difference in time spent in the front half of the tank between fathers exposed to any type of cue compared to control fathers (table 1).

**Table 1.** Results of MCMCglmm models testing predictors of paternal (F0) time spent in the front of the tank and time hiding on days 1, 5 and 10. We tested for potential interactions between visual exposure, olfactory exposure and observation period. We removed any interactions that were not statistically significant. Bold values indicate statistically significant *p*-values.

	mean	95% CI (L, U)	*p*
***time in the front of the tank***			
day 1			
visual exposure	2.08	(0.28, 3.99)	**0.02**
olfactory exposure	−0.23	(−1.85, 1.64)	0.77
observation period	0.36	(−0.88, 1.64)	0.57
length	−0.02	(−0.27, 0.23)	0.87
visual × observation period	−2.7	(−4.59, −0.88)	**0.005**
day 5			
visual exposure	0.48	(−0.63, 1.60)	0.38
olfactory exposure	−0.76	(−1.87, 0.36)	0.16
observation period	−0.004	(−0.37, 0.37)	0.98
length	0.07	(−0.13, 0.28)	0.51
day 10			
visual exposure	−0.38	(−1.10, 0.31)	0.26
olfactory exposure	−0.11	(−0.78, 0.60)	0.74
observation period	−0.05	(−0.56, 0.45)	0.84
length	0.01	(−0.11, 0.14)	0.84

	**mean**	**95% CI (L, U)**	***p***
***time hiding***			
day 1			
visual exposure	0.52	(−2.96, 4.15)	0.77
olfactory exposure	−0.88	(−4.70, 2.83)	0.63
observation period	0.20	(−1.29, 1.69)	0.77
length	0.08	(−0.38, 0.55)	0.76
day 5			
visual exposure	−2.59	(−15.69, 9.72)	0.64
olfactory exposure	−5.00	(−18.19, 6.34)	0.36
observation period	0.05	(−2.84, 2.91)	0.95
length	−1.31	(−4.38, 1.40)	0.29
day 10			
visual exposure	−2.54	(−7.67, 2.21)	0.24
olfactory exposure	−0.86	(−5.69, 3.92)	0.68
observation period	−0.86	(−4.84, 3.04)	0.63
length	0.37	(−0.49, 1.24)	0.36

For time hiding, we did not find that fathers exposed to visual or olfactory cues were spending significantly different amounts of time hiding in the pre- or post-exposure period compared to control fathers across all three days (nonsignificant interaction between paternal treatment and observation period; table 1). We did not detect differences in variance in paternal hiding behaviour (Levene’s test: day 1 pre-period: *F*_3,32_ = 0.44, *p* = 0.72; day 1 post-period: *F*_3,31_ = 0.86, *p* = 0.47) or time spent in the front of the tank (Levene’s test: day 1 pre-period: *F*_3,32_ = 0.78, *p* = 0.51; day 1 post-period: *F*_3,32_ = 1.80, *p* = 0.17) due to paternal treatment.

### 3.2. Offspring activity

We tested offspring of fathers exposed to visual and/or olfactory cues of a predator in an open field assay where we measured exploration (different sections visited) and activity (total sections visited) before and after a predator stimulus, in addition to time frozen immediately after the predator stimulus. All offspring, regardless of paternal treatment, were less active after the predator stimulus compared to before, confirming that offspring behaviourally responded to the predator attack (table 2). There was no effect of paternal treatment on offspring exploration or activity, or freezing behaviour (table 2), although larger offspring were less active and froze for a longer amount of time after the predator stimulus than smaller offspring (table 2).

**Table 2.** Results of MCMCglmm models testing predictors of offspring activity/exploration and freezing behaviour in the open field assay, as well as survival time in the survival assay. We tested for potential interactions between visual exposure, olfactory exposure and observation period where applicable. We removed any interactions that were not statistically significant. Bold values indicate statistically significant *p*-values.

	mean	95% CI (L, U)	*p*
***freezing behaviour***			
visual exposure	−0.39	(−1.30, 0.53)	0.38
olfactory exposure	−0.13	(−1.02, 0.78)	0.78
length	0.17	(0.01, 0.33)	**0.03**

	**mean**	**95% CI (L, U)**	***p***
***activity and exploration***			
*total sections*			
visual exposure	0.07	(−0.34, 0.48)	0.73
olfactory exposure	0.17	(−0.24, 0.58)	0.40
observation period	−0.30	(−0.41, −0.18)	**<0.001**
length	−0.13	(−0.19, −0.06)	**<0.001**
*different sections*			
visual exposure	0.03	(−0.07, 0.14)	0.55
olfactory exposure	0.06	(−0.05, 0.16)	0.27
observation period	−0.03	(−0.10, 0.05)	0.52
length	−0.03	(−0.05, −0.01)	**<0.001**

	**mean**	**95% CI (L, U)**	***p***
***survival***			
visual exposure	−1.11	(−1.91, −0.35)	**0.005**
olfactory exposure	0.08	(−0.72, 0.84)	0.83
length	−0.09	(−0.22, 0.05)	0.21

Variance in offspring freezing behaviour was significantly different across treatment groups (Levene’s test: *F*_3,208_ = 4.00, *p* = 0.008) with offspring of fathers exposed to visual and olfactory cues of predation risk having lower variance than the offspring of fathers exposed to control cues (control offspring standard error: 13.85 s; visual: 12.85 s, olfactory: 10.75 s, visual and olfactory: 5.68 s; electronic supplementary material, figure S1). Similarly, variance in offspring exploration was significantly different in the pre-exposure period (Levene’s test: *F*_3,208_ = 2.96, *p* = 0.03) with lower variance in offspring of fathers exposed to visual and olfactory cues of predation compared to offspring of fathers exposed to control cues (control offspring standard error: 0.36; visual: 0.34; olfactory: 0.44; visual and olfactory: 0.23). There was no difference in variance in the post-exposure period for offspring exploration (Levene’s test: *F*_3,197_ = 1.65, *p* = 0.18). Finally, variance in offspring activity was marginally significant in the post-exposure period of the behavioural assay (Levene’s test: *F*_3,197_ = 2.58, *p* = 0.05), with the lowest variance being in offspring of fathers exposed to visual cues only (control offspring standard error: 4.58; visual: 2.64; olfactory: 3.93; visual and olfactory: 4.41). There was no difference in variance in the pre-exposure period for offspring activity (Levene’s test: *F*_3,208_ = 2.16, *p* = 0.09).

### 3.3. Offspring survival

**Figure 3.**
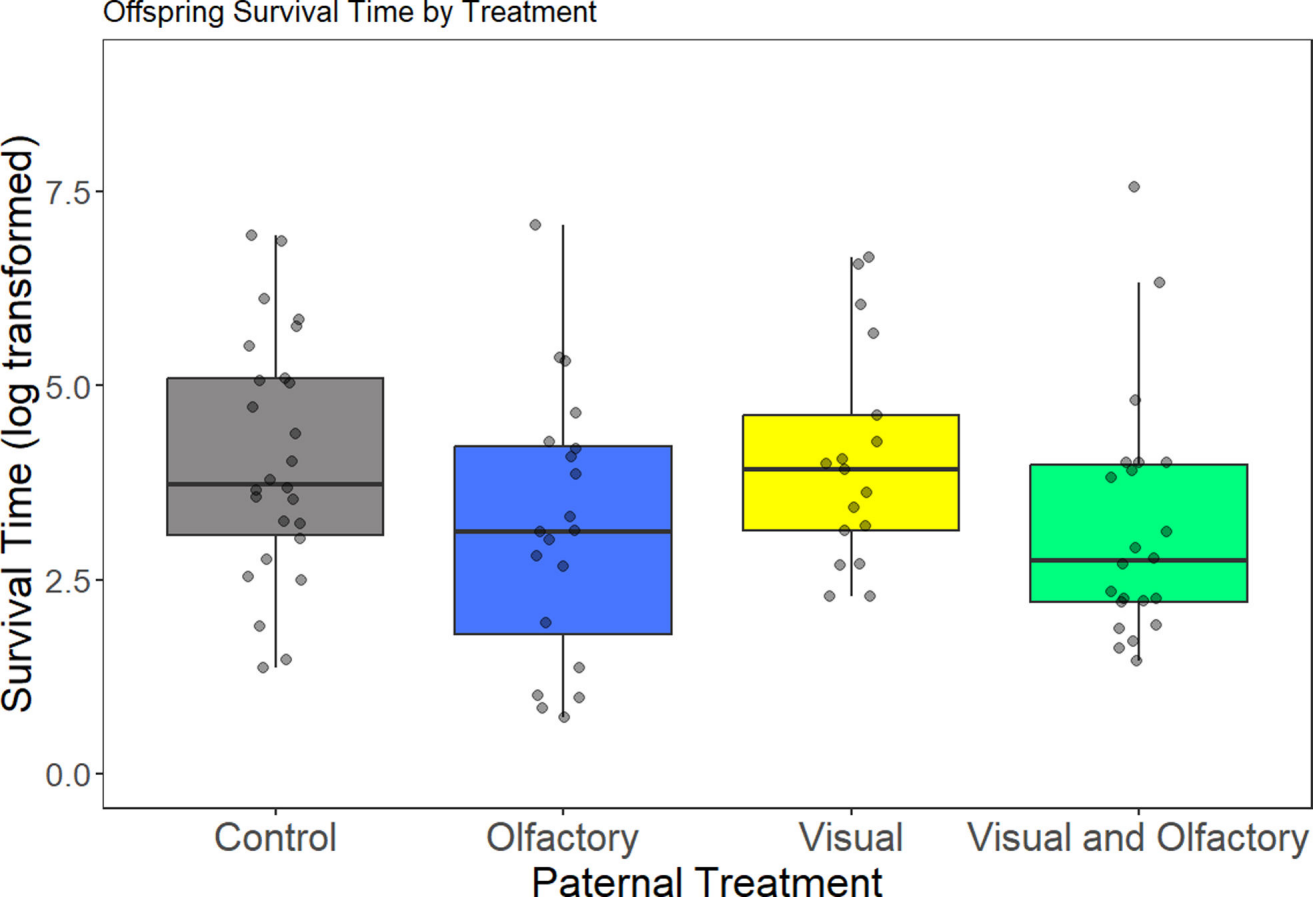
Offspring survival time in the live-predator assay due to paternal treatment (data are median with interquartile range, dots correspond to individuals). We found that the offspring of fathers exposed to the visual cue in the visual-only and visual and olfactory treatment had reduced survival against a live rainbow trout predator. The data for the figure were log-transformed for visualization only and were not transformed in the model for analysing significance.

We measured offspring survival time in pairs against a live predator. We found a significant main effect of paternal visual treatment on offspring survival in the live-predator assay, but no effect of paternal olfactory treatment (table 2). Specifically, the offspring of fathers exposed to visual cues (visual-only and visual and olfactory treatment) were captured faster by the predator compared to offspring of control fathers and fathers that received only olfactory cues (figure 3). Length was not a predictor of survival in the assay (table 2). When we included sex as a covariate in the survival time model, we found a significant three-way interaction between paternal visual exposure, paternal olfactory exposure, and sex (electronic supplementary material, table S3 and figure S2). There were no differences in variance in offspring survival time based on paternal treatment (Levene’s test: *F*_3,81_ = 0.12, *p* = 0.95).

## 4. Discussion

TGP may be a way for parents to prepare their offspring for the future environment, especially in the face of novel predators. We exposed threespined stickleback fathers to either visual, olfactory or both cues of a novel rainbow trout predator. We measured the fathers’ behavioural response before and after exposure to the cue and found that fathers exposed to visual cues (in the visual or visual and olfactory treatment) spent more time in the front half of the tank closer to the predator on the first day of exposure, while we found no significant changes in paternal behaviour in response to olfactory predator cues. We tested the offspring produced by the exposed fathers and found that the offspring of fathers who received visual cues were captured faster by a live rainbow trout predator compared to the offspring of fathers in the control treatment. While these results suggest that paternal exposure to visual cues of a novel predator has consequences for offspring, it does not seem to adaptively prime offspring to cope with novel predators.

Our results agree with other studies demonstrating that many species, including brown anoles, fiddler crabs, and sticklebacks, show a stronger response to visual cues compared to olfactory cues of predators [19,24,41,42]. In our study, fathers spent more time in the front of the tank (and marginally oriented more to the predator; electronic supplementary material, table S2) after exposure to visual cues compared to the pre-exposure period. Although this was not the change in behaviour that we predicted, it is possible that fathers were inspecting the predator, which is a risky behaviour frequently seen in response to predator exposure [24,43] and can allow prey to gain information about the predator [14]. Although the differences in inspection behaviour did not persist past the first day of exposure, this may be because inspection is unnecessary once fathers have already acquired information about the predator.

While fathers responded to visual cues, there was no change in fathers’ behaviour after exposure to olfactory cues compared to the control. Because we fed trout live sticklebacks, our predator cue contained the predator odour of the trout themselves as well as conspecific alarm cues from the ingestion and digestion of the stickleback. There could be several explanations for why fathers did not respond to the olfactory cues. Our results are consistent with previous evidence suggesting that sticklebacks have a weak response to olfactory cues, either of a trout predator or conspecific alarm cue [24]. The weak role of chemical cues could arise because stickleback have poorly developed olfactory organs compared to other fish [44] and because fish with morphological defences (such as armoured sticklebacks) seem to respond strongly to direct predation risk and weakly to chemical cues of predators [45]. It is possible that the lack of response to paternal olfactory cues could be experimental if the concentration of the cue (especially of the conspecific alarm cue component) was not strong enough for fathers to detect it or feel threatened by it. However, other studies that used a similar concentration and volume of olfactory cues saw a change in behaviour due to the olfactory cue only when it was paired with the visual cue [22], or saw a weak effect of olfactory cues alone [24]. In these studies, they measured changes in baseline foraging-related behaviours in response to visual and olfactory cues. Here we sought to capture changes in baseline territory use before and after predator exposure. We would have expected some behaviours (e.g. time spent hiding) to change in response to both visual and olfactory cues. However, other behaviours (e.g. inspection behaviour) may have been less useful in detecting an effect of olfactory cues, especially since our cue was not administered with a continuous flow that would have allowed fathers to detect the source of the cue. Therefore, future studies altering the strength/application of the olfactory cue or measuring additional behavioural (e.g. spine erection, freezing after the application of the cue) or non-behavioural traits (e.g. cortisol levels) would be useful in determining the extent to which sticklebacks attend to olfactory cues.

In addition to changes in paternal behaviour, we found that the offspring of fathers exposed to visual cues showed reduced survival against a live predator in the survival assay. This suggests that the offspring response was correlated with the paternal response: fathers changed their behaviour in response to visual cues, and that is where we saw phenotypic changes in offspring. A positive correlation between parent and offspring responses to predation risk has also been seen in the stress hormone levels of snowshoe hares [46], where the magnitude of maternal response is scaled to offspring phenotypes. This suggests that we may be able to predict the expected offspring behaviour by measuring paternal responses: phenotypic changes in offspring occur primarily when parents recognize and mount a physiological or behavioural response to an environmental cue.

There was no change in survival time in the offspring of fathers exposed to olfactory cues compared to the offspring of control fathers (although see electronic supplementary material). Further, we did not see an additive effect of receiving both cues on the fathers’ behaviour or on the survival of offspring whose fathers received both cues of predation. Instead, offspring whose fathers received both cues were captured just as quickly by the predator as offspring whose fathers received only visual cues. This is perhaps not surprising, given that we found no effect of olfactory cues on their own, possibly because fathers did not detect the olfactory cue (see above). However, it could also suggest that multimodal cues do not improve the detection of predators: having both cues did not indicate a more risky environment or strengthen the antipredator response compared to having one cue. This is consistent with a previous study in tadpoles showing that individuals who received two cues of predation (visual and olfactory) were statistically indistinguishable from individuals who received one cue of predation (olfactory only) [47]. One source of information about the future environment may reach a certain threshold to trigger an antipredator phenotype in offspring, and having more information about the same cue would not influence any further development towards that maximum phenotype [48]. The visual cue may be more reliable because visual cues of predators are likely similar to cues that they have encountered in their evolutionary history [9] and because an individual can assess how close they are to the predator, providing a better spatial and temporal timeline than olfactory cues. A recent meta-analysis found, however, that exposure to multiple cues of predation together did not alter the mean response, but did reduce the variance, i.e. the uncertainty associated with their response [49]. Our results support this: we found that there was lower variance in offspring freezing behaviour and exploration when fathers were exposed to both visual and olfactory cues, relative to other treatment groups. Reduced variance in response to combined cues suggests that while multiple cues can be redundant in terms of mean response, more information about a predator could still be providing more certainty in a biologically significant way.

Offspring of fathers exposed to visual cues were caught faster by the predator than the offspring of fathers who did not receive visual cues. This suggests that, even though the visual cue was enough for fathers to detect and transmit information to offspring, the offspring phenotype was not adaptive for survival. If adaptive parental priming had occurred, we would have expected the offspring of visually exposed fathers to survive longer against a live predator. These maladaptive effects may result from the design of our survival assays, as antipredator behaviour in an artificial arena may not match antipredator behaviour in the wild. However, other studies using different experimental designs have also found reduced offspring survival in live-predator assays when parents were exposed to a novel predator cue [50]. There are several ultimate explanations for why we might see maladaptive TGP in our study, despite the fact that maladaptive TGP should be selected against.

First, these maladaptive effects may arise because even if parents have the ability to detect novel cues, the cue does not necessarily provide information on how to optimally respond to it [9]. In situations where ancestral pathways for TGP are co-opted to respond to novel cues, an inappropriate phenotype in offspring could be triggered. For example, olfactory cues of herbicides are similar to olfactory cues of predators; therefore, exposure to herbicide erroneously leads to antipredator phenotypes [51]. However, reduced survival in offspring of predator-exposed fathers has also been seen with cues from a native predator [29]. Second, survival may trade off with other life-history traits, such that paternal predator exposure decreases survival in offspring while promoting other fitness-related traits at other life stages (e.g. increased boldness that leads to higher reproductive success). This was seen in a marine invertebrate, where maternal exposure to copper increased copper resistance in her offspring, but it reduced their survival [52]. Although we did not see any changes in behaviour in our other assay (e.g. increased boldness or activity in the open field assay), other studies in stickleback have found that paternal exposure to predation risk resulted in offspring that were less anxious and had increased risk-taking behaviour [29,30]. Third, previous studies have demonstrated that short parental exposures might be more likely to lead to maladaptive effects than longer exposures because longer exposures provide more time for parents to acclimate to the cue [53,54]. For example, adult sheepshead minnows exposed to elevated temperatures for 7 days did not see beneficial growth in offspring reared at that temperature, but when adults were exposed for 30 days, the growth rate of offspring increased by 32% [55]. An exposure time longer than 10 days may be needed for fathers to be able to process and adapt to the novel predator cue in ways that have fitness benefits for offspring. Finally, it is possible that maladaptive effects arise because of the integration of maternal and paternal cues. Previous studies have found that survival against predators was reduced in offspring of predator-exposed fathers and predator-naive mothers (similar to the offspring in our experiment), but not in offspring that had both a predator-exposed mother and father [29]. This suggests that offspring fitness is highest when parental cues match (either both parents are predator-naive or both are predator-exposed), but that offspring face fitness deficits when paternal cues do not match maternal cues (paternal cues of predation risk, maternal cues of safety). Future work should focus on how the integration of maternal and paternal effects can influence both the magnitude and adaptive significance of transgenerational effects for offspring.

In conclusion, we showed that paternal exposure to visual cues, but not olfactory cues, elicited behavioural changes in the fathers; this within-generational response correlated with a reduction in survival in offspring of fathers who received visual cues from novel predators. Although parents recognize and respond to novel cues in ways that can be passed to offspring, we found that these effects can be maladaptive for offspring. Maladaptive TGP in response to novel stressors could result in inappropriate behaviours in the presence of the stressor, or a failure to respond at all. This increases the likelihood of a mismatch between offspring phenotype and the environment because parents are unable to predict the future environment to ‘prime’ their offspring. However, previous studies have found that adaptive transgenerational responses to novel cues can be dependent on the context in which parents are exposed to the novel cue [29,54]; for example, an adaptive response in our study may have been elicited with a longer exposure time or a consideration of how maternal experience interacts with the paternal experience. Generally, because individual phenotypes result from the integration of multiple sources of information and multiple types of stressors, paternal experiences cannot be considered in isolation. Future research predicting adaptive responses to environmental stressors should consider how these other factors (e.g. social interactions with conspecifics) influence the adaptive nature of paternal effects in rapidly changing environments.

## Data Availability

All data and code are uploaded as electronic supplementary material [56].
